# Association of an Electronic Health Record Add-on App for Neonatal Bilirubin Management With Physician Efficiency and Care Quality

**DOI:** 10.1001/jamanetworkopen.2019.15343

**Published:** 2019-11-15

**Authors:** Kensaku Kawamoto, Polina Kukhareva, Julie H. Shakib, Heidi Kramer, Salvador Rodriguez, Phillip B. Warner, David Shields, Charlene Weir, Guilherme Del Fiol, Teresa Taft, Carole H. Stipelman

**Affiliations:** 1 Department of Biomedical Informatics, University of Utah, Salt Lake City; 2 Department of Pediatrics, University of Utah, Salt Lake City

## Abstract

**Importance:**

The usefulness of electronic health record (EHR) systems could be significantly enhanced by innovative, third-party EHR add-on apps.

**Objective:**

To evaluate whether an EHR add-on app for neonatal bilirubin management can save clinicians time and improve patient care.

**Design, Setting, and Participants:**

This quality improvement study was conducted at the University of Utah Health Well Baby nursery and outpatient clinics and consisted of 4 substudies: (1) time savings were estimated in an experimental task-timing study comparing the time required for physicians to manage newborns’ bilirubin levels with and without the add-on app, (2) app use was estimated from app logs, (3) health care use measures and guideline compliance were compared retrospectively before and after the intervention, and (4) clinician-perceived usability was measured through System Usability Scale surveys. The study took place between April 1, 2016, and September 3, 2019. Data analyses were conducted from October 30, 2018, to September 23, 2019.

**Interventions:**

At baseline, clinicians used a manual approach to ensure compliance with an evidence-based clinical guideline for neonatal bilirubin management. To facilitate guideline compliance, an EHR add-on app that automatically retrieves, organizes, and visualizes relevant patient data was developed. The app provides patient-specific assessments and recommendations, including the risk of rebound hyperbilirubinemia following phototherapy based on a predictive model. The add-on app was integrated with the University of Utah Health EHR on April 12, 2017.

**Main Outcomes and Measures:**

Clinician time savings, app use, health care use measures, guideline-compliant phototherapy ordering, and perceived usability as measured by the System Usability Scale survey. The survey is composed of 10 statements with responses ranging from 1 (strongly disagree) to 5 (strongly agree). The survey results in a single score ranging from 0 to 100, with ratings described as worst imaginable (mean System Usability Scale score, 12.5), awful (20.3), poor (35.7), okay (50.9), good (71.4), excellent (85.5), and best imaginable (90.9).

**Results:**

In 2018, the application was used 20 516 times by clinicians for 91.84% of eligible newborns. Use of the app saved 66 seconds for bilirubin management tasks compared with a commonly used tool (95% CI, 53-79 seconds; *P* < .001). Following the intervention, health care use rates remained stable, while orders for clinically appropriate phototherapy during hospitalization increased for newborns with bilirubin levels above the guideline-recommended threshold (odds ratio, 1.84; 95% CI, 1.16-2.90; *P* = .009). Surveys indicated excellent usability (System Usability Scale score, 83.90; 95% CI, 81.49-86.31).

**Conclusions and Relevance:**

Well-designed EHR add-on apps may save clinicians time and improve patient care. If time-saving apps, such as the bilirubin app, were implemented widely across institutions and care domains, the potential association with improved patient care and clinician efficiency could be significant. The University of Utah Health bilirubin app is being prepared for release into EHR app stores as free-to-use software.

## Key Points

**Question:**

Is an electronic health record add-on app for neonatal bilirubin management associated with time savings for clinicians and improved quality of care?

**Findings:**

In this quality improvement study, an electronic health record add-on app for neonatal bilirubin management saved clinicians a mean of 66 seconds for bilirubin management tasks compared with a commonly used tool. In a retrospective pre-post analysis, the odds of clinically appropriate phototherapy orders during hospitalization increased significantly by 84%.

**Meaning:**

These findings suggest that well-designed electronic health record add-on apps may be associated with time savings for physicians and improvements in patient care.

## Teaser

This quality improvement study evaluates whether an electronic health record add-on app for neonatal bilirubin management is associated with clinician time savings and improvements in patient care.

## Introduction

Despite billions of dollars invested, electronic health records (EHRs) often fall short in supporting efficient, high-quality patient care.^1^ Ambulatory care physicians can spend 2 hours on EHR and desk work for every hour spent in direct clinical face time,^2^ and inpatient physicians can spend 5 hours on such tasks for every hour spent in direct patient care.^3^ In a statewide survey of Rhode Island physicians conducted in 2017, close to two-thirds of physicians reported that use of EHRs added to their daily frustration.^4^

Through emerging technology standards, EHRs now allow the integration of add-on apps, whereby third-party app developers can deliver innovative solutions that enhance the utility of EHRs for targeted health care tasks and domains.^5,6^ Just as smartphones deliver a better user experience through a variety of add-on apps, the hope is that EHRs can deliver improved user experiences through such apps. Unlike EHR vendors, who may be overwhelmed with competing priorities, such as maintaining legacy functionality and providing support to thousands of customers for a wide range of clinical and administrative tasks, a third-party app developer can focus entirely on optimizing a comparatively small range of decision tasks. Apps also use a technical framework designed to facilitate substituting one app for another, whereas switching EHRs is a disruptive and costly process. Thus, app developers may be inherently incentivized to optimize patient care and the user experience in their domain of focus, whereas EHR vendors are unlikely to gain or lose a customer based on their level of support for any specific decision task. Also, unlike EHR vendors, who may have years-long cycles for feature prioritization and development, third-party app developers can adopt a much more rapid and iterative development approach, with the ability to develop and release enhancements much more quickly based on user feedback.

The technology enabling EHR add-on apps is known as Substitutable Medical Applications and Reusable Technologies on Fast Healthcare Interoperability Resources (SMART on FHIR; pronounced *smart on fire*).^7^ Apps enabled by SMART on FHIR use the Health Level Seven International (HL7) SMART standard^8^ to enable single sign-on and integration with the EHR user interface, as well as the HL7 FHIR data interface standard^9^ to pull in relevant patient data automatically. Although the evidence of the influence of SMART on FHIR apps on clinical outcomes is limited, investigators at Boston Children's Hospital showed in 2017 that the introduction of a SMART on FHIR app for pediatric blood pressure visualization was associated with an increase in the recognition of abnormal blood pressure (7.1% vs 4.9%; *P* < .001).^10^

The present study reports on an evaluation of an EHR add-on app that was implemented in 2017 at an academic medical center to support the American Academy of Pediatrics (AAP) guideline on the management of neonatal hyperbilirubinemia.^11^ To manage newborns’ care according to this guideline, the clinician must retrieve disparate data scattered across the medical record, synthesize the data for risk classification, and apply guideline algorithms to identify patient-specific care needs, such as the administration of phototherapy when bilirubin levels exceed risk-based treatment thresholds.^11^ The app was designed to support these tasks by retrieving relevant data, providing a visual data summary, and delivering guideline-based recommendations on next steps. We also designed the app to support known success factors for clinical decision support (CDS) systems, including providing CDS at the time and location of decision-making, providing recommendations rather than just assessments, integrating with the EHR, and minimizing the need for additional clinician data entry.^12^ The goal of this study was to evaluate our hypothesis that this EHR add-on app would save clinicians time and improve care quality.

## Methods

### Study Design

This was a mixed-methods quality improvement study that included 4 separate substudies: (1) an experimental task-timing study to estimate time savings, (2) an observational study of app use, (3) a retrospective pre-post intervention study evaluating patient outcomes, and (4) a usability survey study. App implementation and the subsequent quantitative evaluations of app use and patient outcomes were exempted as quality improvement by the University of Utah Institutional Review Board (IRB). The timing study and survey were approved by the IRB, and participants consented verbally after reading an IRB-approved informed consent cover letter. This study report follows the Revised Standards for Quality Improvement Reporting Excellence (SQUIRE 2.0) reporting guidelines.^13^

The study was conducted at University of Utah Health (UUH), an academic health care system using the Epic EHR (EPIC Systems Corp). Inpatient care for well newborns is provided in a newborn nursery as mother-baby couplet care. Follow-up visits are conducted in 27 UUH clinics. Universal bilirubin screening was implemented in the nursery on March 31, 2016. Prior to the UUH app intervention, a stand-alone web-based tool known as BiliTool (BiliTool Inc) was used for bilirubin management.^14^

Newborns born at 35 weeks’ gestation or longer and admitted to the nursery were included in the analysis. Follow-up visits at UUH clinics within 14 days from discharge were included.

Before proceeding with the design and development of the Bili App, and in accordance with UUH’s standard operating procedures for new app development, the project team explored whether native EHR approaches could be used to meet the user needs. In particular, conventional EHR mechanisms for CDS, such as alerts, reminders, and order sets, were considered. However, it was determined that these conventional CDS mechanisms would not be able to support the desired functionality, including providing a graphical summary of relevant patient data, supporting the full complexity of the underlying clinical decision logic, and adjusting recommendations based on user input. Thus, the decision was made to proceed with app development.

The app leverages the SMART on FHIR framework. Starting from a basic app developed by Intermountain Healthcare to graph bilirubin levels against a risk nomogram,^15,16^ the University of Utah ReImagine EHR team iteratively refined the app based on physician feedback. The ReImagine EHR team includes clinical informaticists with expertise in areas including software development, software architecture, standards-based interoperability, cognitive psychology, and biostatistics. Both the SMART and a core set of FHIR data standards, known as the US Core FHIR profiles,^17^ were natively supported by the Epic EHR, facilitating integration of the app. However, to achieve the full set of functionality requested by clinicians, custom FHIR interfaces had to be developed to pull additional data elements, including the mother’s laboratory data and outpatient phototherapy orders. Such custom FHIR interfaces can be shared across health care systems and may become unnecessary as EHR vendors increase the scope of data covered by their native FHIR data interfaces.

The resulting app (Figure) supports neonatal bilirubin management according to the AAP guideline^11^ and estimates the risk of postphototherapy rebound hyperbilirubinemia.^18^ The app was deployed institution-wide on April 12, 2017. Beyond a brief email message to EHR users on app availability, app awareness was spread by word-of-mouth.

**Figure.**
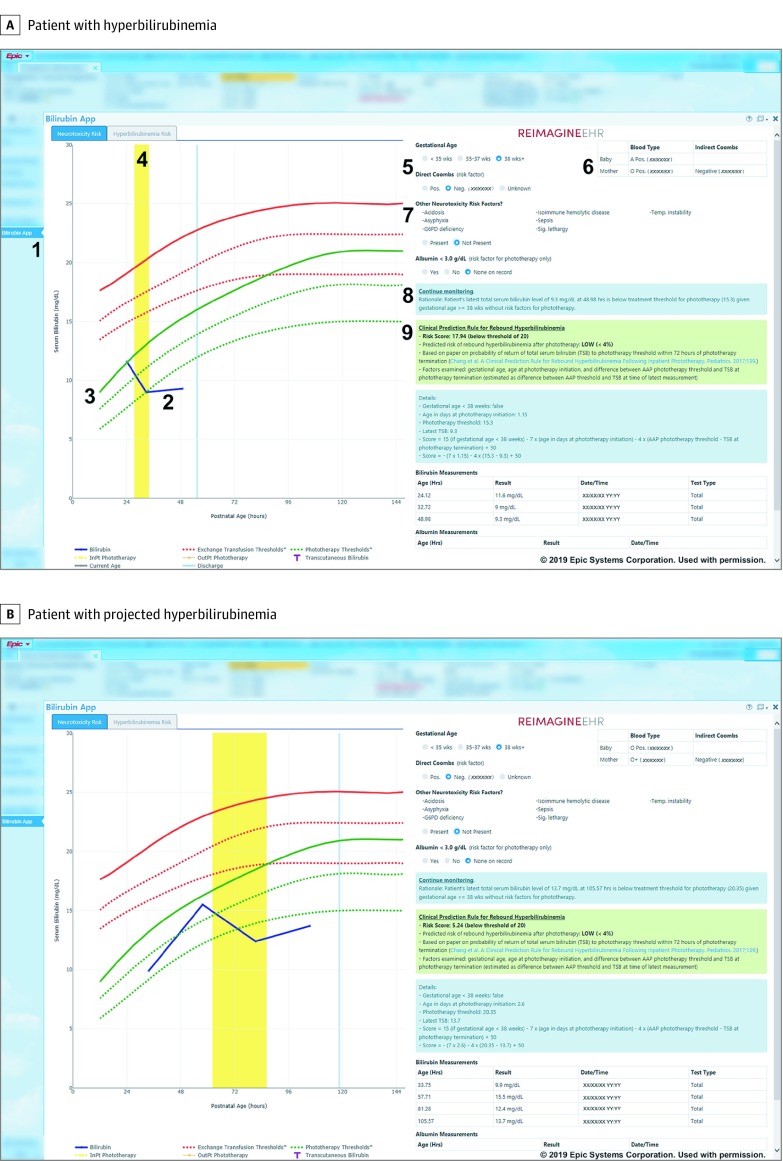
App Screenshots for Bilirubin Analysis Screenshots shown for a patient with hyperbilirubinemia (A) and projected hyperbilirubinemia (B). 1, link to open the app in the EHR sidebar; 2, bilirubin levels over time (blue line); 3, patient-specific phototherapy threshold according to American Academy of Pediatrics guideline (green line); 4, inpatient phototherapy (yellow area); 5, patient-specific risk factors; 6, mother’s laboratory information; 7, other neurotoxicity risk factors; 8, guideline-based care recommendations (in blue box); and 9, predicted risk of rebound hyperbilirubinemia following phototherapy (in green box). AAP indicates American Academy of Pediatrics; InPt, inpatient; Neg, negative; OutPt, outpatient; Pos, positive; TSB, total serum bilirubin. Used with permission from Epic Systems Corp.

Resident physicians screen for and manage hyperbilirubinemia in the nursery under the guidance of attending physicians. Prior to the introduction of the UUH app, clinicians used the BiliTool website, which is available as a link in the EHR. Clinicians would open the website and manually enter the time of birth as well as the last total bilirubin level and associated specimen collection time. The website takes these inputs and provides recommendations for all 3 potential risk levels as defined by the AAP guideline (lower, medium, and higher risk).^11^ BiliTool does not calculate the patient-specific risk level; as such, clinicians independently assessed the patient’s risk status using data in the EHR, with the patient’s risk level determined primarily by the patient’s gestational age and direct Coombs test results.^11^

With the UUH app, clinicians launch the app through the EHR’s sidebar (Figure, A, item 1). The app then loads as any other EHR screen does. There is no need for additional login and all the information is pulled automatically from the EHR. If the patient does not have direct Coombs test results, the app prompts the clinician whether other neurotoxicity risk factors are present before providing a recommendation (Figure, A, item 7). Clinicians then review the patient data, including the patient’s bilirubin levels over time (Figure, A, item 2); the patient’s risk-specific threshold where phototherapy is indicated (Figure, A, item 3); history of phototherapy administration (Figure, A, item 4); other relevant data, including the mother’s blood type and indirect Coombs test results (Figure, A, item 6); patient-specific recommendations on next steps (Figure, A, item 8); and, if applicable, the patient’s estimated risk of rebound hyperbilirubinemia following phototherapy (Figure, A, item 9). Clinicians can also evaluate the influence of potential changes on patient risk factors, such as if the patient’s gestational age at birth was 37 weeks 6 days rather than 38 weeks 0 days (Figure, A, item 5).

### Substudies

In a time-savings evaluation, 12 pediatric and family medicine resident physicians on service in the nursery (including 7 interns, 3-second-year residents, and 2 third-year residents) were recruited to complete bilirubin management tasks for their current patients (n = 42 patients). Each resident physician completed these bilirubin management tasks for 2 to 5 patients, with every patient randomly assigned to either Bili App or BiliTool use. Participants were asked to complete their work as they usually would for making a decision regarding the patients' care needs with regard to hyperbilirubinemia screening and management. Typically, this process involved reviewing the bilirubin levels, identifying risk factors, and making a decision about appropriate next steps, including possible additional bilirubin testing, phototherapy use, and discharge. We recorded the EHR screen as the residents completed these bilirubin management tasks. Participants were asked to act as if the observer was not present. Specifically, participants were asked to not “think aloud” or describe what they were doing while performing these tasks.

Task time was calculated from screen recordings from the moment the patient’s medical record was opened in the EHR to the moment when residents indicated that they were finished. Time savings were estimated using generalized linear regression with generalized estimating equations to account for correlation within timings from the same residents. We included subset analyses for users with different experience levels.

Based on EHR logs for eligible newborns born in 2018, app use was measured as the number of uses and the proportion of newborns with admissions (n = 3826) and follow-up visits (n = 1932) for whom the Bili App was used. To calculate annual time savings for UUH, we multiplied the number of uses in 2018 by the mean time savings from the task-timing study.

The preintervention period was April 1, 2016, through March 31, 2017 (n = 3714), and the postintervention period was May 1, 2017, through April 30, 2019 (n = 7520), excluding 5 newborns with missing gestational age or sex. There were no other interventions related to bilirubin management introduced in the nursery during the study timeframe.

Reported patient characteristics include sex, gestational age less than 38 weeks, direct Coombs test results, and performance of a serum bilirubin test. Patient characteristics before and after the intervention were compared using χ^2^ tests. Health care use measures were length of stay, intensive care unit admissions, urgent care visits, and readmissions. Risk-specific thresholds for phototherapy (Figure, A, item 3) were determined at 12 to 144 hours of life based on whether the gestational age was less than 38 weeks and whether a positive direct Coombs test result was documented.^11^ The potential existence of other risk factors was not assessed for these purposes. Phototherapy use was evaluated for 3 mutually exclusive patient subsets based on whether patients (1) had a bilirubin level that was above the phototherapy threshold (hyperbilirubinemia, n = 591), (2) had a bilirubin level that was projected from the rate of rise to be above the threshold within 24 hours (projected hyperbilirubinemia, n = 557) (Figure, B, provides an example), or (3) met neither condition (nonhyperbilirubinemia, n = 9980). For patients with hyperbilirubinemia, we evaluated phototherapy ordering rates within 4 hours of the first documented serum bilirubin level result above the AAP phototherapy threshold during hospitalization. For patients with projected hyperbilirubinemia or nonhyperbilirubinemia, we evaluated phototherapy ordering rates for any time during hospitalization.

Generalized linear models were used for the evaluation of all measures. Gamma regression was used for length of stay and logistic regression was used for all other measures. Covariates included in the model were gestational age less than 38 weeks, positive direct Coombs test results, and, for the phototherapy-ordering measures for patients with projected hyperbilirubinemia or nonhyperbilirubinemia, the shortest distance between a bilirubin level and the phototherapy threshold. Estimated marginal means and percentages were calculated at mean covariate values. Odds ratios and fold increases were estimated by exponentiating the regression coefficients. Percentage changes in odds were calculated as the odds ratio −1, then multiplied by 100.

In February 2019, an invitation to participate in a System Usability Scale (SUS) survey was emailed to 208 clinician users who had used the Bili App in January 2019; of these, 109 users (52.40%) participated.^19^ The SUS survey is composed of 10 statements with responses ranging from 1 (strongly disagree) to 5 (strongly agree). The survey results in a single score ranging from 0 to 100. Bangor et al^20^ described the adjective ratings associated with SUS scores: worst imaginable (mean SUS score, 12.5), awful (20.3), poor (35.7), okay (50.9), good (71.4), excellent (85.5), and best imaginable (90.9).

### Statistical Analysis

Data for analyses were retrieved from the enterprise data warehouse. All statistical analyses were performed using R, version 3.5.1 (R Foundation) and 2-tailed tests. *P* values <.05 were considered significant. The study took place between April 1, 2016, and September 3, 2019. Data analyses were conducted from October 30, 2018, to September 23, 2019.

## Results

The Bili App required a mean of 35 seconds (95% CI, 27-42 seconds) to complete the bilirubin management tasks compared with 100 seconds (95% CI, 89-112 seconds) with a commonly used tool (66-second time savings, 95% CI, 53-79 seconds; *P* < .001) (Table 1). Interns saved 81 seconds, while second and third-year residents saved 56 seconds.

**Table 1.** Randomized Task-Timing Study: Time Required to Complete Bilirubin Management Tasks

Physician Group	No.		Time, Estimated Marginal Mean (95% CI), s			*P* Value^a^
	Patients	Residents	BiliTool Time	UUH App Time	Time Savings	
All resident physicians	42	12	100 (89-112)	35 (27-42)	66 (53-79)	<.001
Interns	21	7	117 (99-134)	35 (24-47)	81 (64-98)	<.001
Second- and third-year residents	21	5	88 (82-94)	32 (29-35)	56 (49-63)	<.001

^a^ *P* values are based on linear regression using generalized estimating equations to account for within-physician correlation.

In 2018, the app was used 20 516 times (Table 2), including 17 812 times for 91.84% of births in the inpatient setting and 2704 times in outpatient settings for 39.18% of patients with follow-up visits. The app was accessed by 77 fellows and attending physicians, 107 residents, 162 registered nurses, 136 medical students, and 45 other clinicians, such as nurse practitioners and physician assistants (Table 2). Given that the app was used 20 516 times overall, we estimated 374.36 (95% CI, 301.19-447.59) hours of time savings annually for all clinicians at UUH, including 235.12 (95% CI, 189.16-281.11) hours of time savings for residents.

**Table 2.** App Use Study: Number of Uses and Estimated Time Savings per Year in 2018^a^

Clinician Role	No. of Clinicians	Uses per Clinician, Mean (95% CI)	Sum of Uses per Role	Estimated UUH Time Savings (95% CI)
All clinicians	527	38.93 (32.78-45.08)	20 516	374.36 (301.19-447.59)
Physician				
Resident	107	120.42 (100.18-140.66)	12 885	235.12 (189.16-281.11)
Fellow and attending	77	51.77 (34.35-69.19)	3986	72.73 (58.52-86.96)
Registered nurse	162	13.25 (9.86-16.64)	2146	39.16 (31.50-46.82)
Medical student	136	8.34 (7.56-9.12)	1134	20.69 (16.65-24.74)
Other	45	8.11 (3.49-12.73)	365	6.66 (5.36-7.96)

Abbreviation: UUH, University of Utah Health.

^a^ Time savings are estimated based on an assumption that users saved 66 (95% CI, 53-79) seconds per use.

Patient characteristics and outcomes pre-post intervention are summarized in Table 3. There were no significant changes in patient characteristics and health care use measures. For patients with hyperbilirubinemia, the adjusted percentage of newborns for whom phototherapy was ordered within 4 hours of the first documented inpatient serum bilirubin test result above the AAP treatment threshold increased significantly from 74.27% to 84.12% (odds ratio [OR], 1.84; 95% CI, 1.16-2.90; *P* = .009). For patients with projected hyperbilirubinemia, the adjusted percentage of newborns for whom phototherapy was ordered increased significantly from 27.34% to 53.41% (OR, 3.05; 95% CI, 2.01-4.62; *P* < .001). For patients without hyperbilirubinemia, the adjusted percentage of newborns for whom phototherapy was ordered remained stable (OR, 0.95; 95% CI, 0.68-1.33; *P* = .78).

**Table 3.** Retrospective Pre-Post Intervention Study: Patient Characteristics and Outcomes

Variable	Preintervention	Postintervention	OR/Fold Increase	*P* Value
**Patient Characteristics, No. (%)**^a^				
Eligible newborns	3714	7520	NA	
Female	1875 (50.48)	3737 (49.69)	NA	.43
Gestational age <38 wk	662 (17.82)	1443 (19.19)	NA	.08
Newborns with positive direct Coombs test result	303 (8.16)	550 (7.31)	NA	.11
Newborns with serum bilirubin test result	3678 (99.03)	7450 (99.07)	NA	.84
**Patient Outcomes, Estimated Marginal Mean (95% CI)**^b^				
Health care use measures^c^				
Length of stay after birth, d	3.12 (3.00-3.25)	3.08 (2.97-3.19)	0.99 (0.95-1.02)	.43
Intensive care unit admission, %	8.51 (7.25-9.97)	9.36 (8.22-10.63)	1.11 (0.94-1.31)	.21
Urgent care visit, %	2.58 (1.95-3.4)	2.97 (2.36-3.74)	1.16 (0.90-1.49)	.25
Readmission, %	10.71 (9.34-12.26)	10.69 (9.51-11.99)	1.00 (0.87-1.15)	.97
Phototherapy ordering, %				
Patients with hyperbilirubinemia (n = 591)^c^	74.27 (66.51-80.75)	84.12 (80.11-87.45)	1.84 (1.16-2.90)	.009
Patients with projected hyperbilirubinemia (n = 557)^d^	27.34 (20.36-35.65)	53.41 (46.44-60.25)	3.05 (2.01-4.62)	<.001
Patients without hyperbilirubinemia (n = 9980)^d^	0.25 (0.16-0.39)	0.24 (0.15-0.37)	0.95 (0.68-1.33)	.78

Abbreviations: NA, not applicable; OR, odds ratio.

^a^ *P* values based on χ^2^ tests.

^b^ *P* values based on multivariate regression.

^c^ Values adjusted for gestational age less than 38 weeks and positive direct Coombs test results.

^d^ Values adjusted for the gestational age less than 38 weeks, positive direct Coombs test results, and distance of the bilirubin level from the phototherapy threshold.

The mean SUS score indicated excellent usability (SUS, 83.90; 95% CI, 81.49-86.31) (Table 4).^20^ Attending physicians rated the system at 91.05 (95% CI, 86.31-95.79), resident physicians at 86.56 (95% CI, 82.14-90.98), registered nurses at 81.63 (95% CI, 81.63 77.85-85.41), and medical students at 76.25 (95% CI, 65.25-87.25). The benchmark mean SUS score across industries is 68.^21^ The app was recognized with multiple awards from the Department of Health and Human Services’ Provider User-Experience Challenge for EHR-integrated apps.^22^

**Table 4.** Usability Survey Study: SUS Scores^a^

Clinician Role	No. of Clinicians	SUS (95% CI)
All clinicians	109	83.90 (81.49-86.31)
Physician		
Resident	24	86.56 (82.14-90.98)
Attending	19	91.05 (86.31-95.79)
Registered nurse	49	81.63 (77.85-85.41)
Medical student	8	76.25 (65.25-87.25)
Other	9	80.83 (69.58-92.08)

Abbreviation: SUS, System Usability Scale.

^a^ The SUS survey is composed of 10 statements with responses ranging from 1 (strongly disagree) to 5 (strongly agree). The survey results in a single score ranging from 0 to 100. Bangor et al^20^ described the adjective ratings associated with SUS scores: worst imaginable (mean SUS score, 12.5), awful (20.3), poor (35.7), okay (50.9), good (71.4), excellent (85.5), and best imaginable (90.9).

## Discussion

Electronic health records are evolving into platforms in which third-party apps add value to their users through integrated tools targeted at specific decisions and tasks. This study may provide empirical support for this vision, in which an add-on app for neonatal bilirubin management was widely used, was associated with clinicians time savings and improved guideline-compliant care, and had high perceived usability. To our knowledge, this is one of the first studies to provide real-world, long-term data suggesting that EHR add-on apps using the emerging SMART on FHIR standard can save clinicians time, improve care, and provide a positive user experience.

The app focuses on a narrow domain and saves only a minute per use, and yet the potential for time savings if deployed on a national scale is significant. If extrapolated to the approximately 3.4 million nonpreterm births annually in the United States,^23^ universal use of the app could potentially save more than 300 000 hours of clinician time every year. Given the limited extent to which EHRs currently support task-optimized data retrieval and synthesis, there are likely millions of hours of clinician time each year that could be saved through the widespread deployment of similar EHR add-on apps developed through a user-centered design process.

Beyond the time savings, another finding was the improvement in appropriate phototherapy ordering. Studies have shown that patients often do not receive recommended care.^24,25^ Moreover, these challenges in care quality may be associated with the time constraints that clinicians face; Yarnall et al^26^ found, for example, that just satisfying US Preventive Services Task Force A and B recommendations would require 7.4 hours per working day of physician time. By making it easier to provide appropriate care, tools such as the app could engender a positive cycle of clinician time savings and improved patient care.

Through the University of Utah ReImagine EHR initiative, we now have experience with implementing a number of SMART on FHIR apps in addition to the bilirubin app. Based on this experience, we believe that several specific features of the bilirubin app have helped to make this implementation successful. These system features include those identified in a meta-analysis of the literature as being important to CDS success: provision of CDS at the time and location of decision-making, provision of recommendations rather than just assessments, integration with the EHR, and the minimal need for additional clinician data entry.^12^ We believe our findings are also congruent with systematic reviews of information displays for critical care, which found that comprehensive information displays that integrate information from multiple sources (eg, laboratory test results, procedures) and present trend data graphically had the strongest association with positive clinician performance and patient outcomes, possibly owing to improved pattern recognition and situational awareness.^27,28^ By automating low-level cognitive tasks, such as retrieving, organizing, sorting, and graphically representing data, integrated information displays let clinicians spare valuable cognitive resources for high-level and complex cognitive tasks. For example, the significantly higher odds of phototherapy orders among patients with projected hyperbilirubinemia suggest that the app may help clinicians to more efficiently identify patients for whom phototherapy may be indicated (Figure, B). Our findings are consistent with cognitive research on working memory, which has found that cognitive load can be reduced—and user performance improved—through displays that match users’ mental models^29^ and automate subtasks, such as information searching, that distract and reduce working memory.^30^

In addition, we believe key factors in the app’s widespread adoption are the reduction in cognitive effort and associated time savings, which can provide a relief from the burden otherwise imposed by EHRs.^2,3^ Because the app applies to practically every newborn, its use became almost universal in the newborn nursery. As such, the app was able to automatically provide CDS as a part of routine clinician workflow, which is a factor that has repeatedly been found to be critical to CDS success.^31,32^

We are actively working to disseminate the app as a free tool through EHR app stores and hope to report on the adoption and outcome of the app’s use at other institutions. We are also developing numerous additional EHR add-on apps through the University of Utah ReImagine EHR initiative to optimize patient care and the clinicians’ EHR experience. Further research is also needed on how to best support clinical decision-making through EHR add-on apps, such as through the application of cognitive science principles and CDS best practices.

### Limitations

This study has limitations. Although the app uses a standards-based approach that should be deployable across health care systems and EHR products, this was a single-center study, and further studies are needed to demonstrate generalizability. The pre-post intervention design could be confounded by secular trends; however, we used multivariate, generalized linear models to adjust for covariates. Also, for the health care use measures (length of stay, intensive care unit admission, urgent care visit, and readmission), we did not limit our analyses to events related to hyperbilirubinemia (eg, intensive care unit admissions due to hyperbilirubinemia). Conversely, the task-timing study was limited to inpatient residents, such that overall time savings may differ from our estimates if other types of clinicians save more or less time from use of the app. Another limitation is that local users were actively involved in the development of the intervention—a factor known to correlate with CDS success^12^ and thus potentially overestimate the outcome that can be expected at other institutions. However, most users of the app are residents, who rotate in and out of the nursery service on a regular basis and had no involvement in the development of the app. In addition, while EHR add-on apps are a promising solution to support clinicians in complex cognitive tasks, they are unlikely to replace more transactional EHR functionality that is tightly coupled with the EHR, such as order entry. Where possible, native EHR functionality should be improved to preserve a cohesive user experience. To this end, as EHR add-on apps grow in their prevalence, it will become increasingly important for SMART on FHIR apps and the underlying EHRs to adopt common user interface conventions.

## Conclusions

The findings of this study suggest that well-designed EHR add-on-apps can save clinicians time, improve care, and enhance the EHR user experience by supporting complex decision tasks. Further research is needed to evaluate the generalizability of these findings in other health care domains and settings. If well-designed EHR add-on apps were widely implemented across health care organizations, the potential for improved patient care and clinician efficiency could be significant.
